# Managing HAM/TSP with Fampridine

**DOI:** 10.1186/1742-4690-11-S1-P38

**Published:** 2014-01-07

**Authors:** M Marcio Menna-Barreto

**Affiliations:** 1 HTLV Regional Reference Center, Porto Alegre Municipal Prefecture, Porto Alegre, RS, Brazil

## 

HTLV-I and HTLV-II are endogenous and have shown high prevalence rates among blood donors (1.4%), pregnant women (1.1%), Guarani amerindians (5.8%), HIV-HTLV-I/HTLV-II coinfected patients (16.3%) and spastic paraparetic individuals (21.6%) among indivividuals living in the city of Porto Alegre, southern Brazil. Tropical spastic paraparesis/HTLV-I associated myelopathy is a immune-mediated inflammatory disease in mid-thoracic spinal cord determining a myelopathy, with spastic-ataxic gait, intestinal and micturitional disorders. No medication was proved to be a disease modifying drug, just physical therapy and symptomatic management. No antiretroviral drug could benefit the patients. Fampridine, a selective neuronal potassium channel blocker, was licenced in USA and other countries, since April 2010, for the treament of gait disorders in Multiple Sclerosis. Some clinical trials have shown some benefit in patients with other kind of spinal cord diseases. Since mid-2012 seven patients monoinfected by HTLV-I and HAM/TSP are regularly using. Six of them improved in ataxia, gait and strengh in the lower limbs. Micturictional dysfunction improved in two. This off-label observational experience offer a preliminary data for a broad discussion.

